# Single incision laparoscopic approach for infected necrotizing pancreatitis: A case report

**DOI:** 10.1016/j.ijscr.2020.07.020

**Published:** 2020-07-15

**Authors:** N. den Dekker, A.A.J. Grüter, S.E. van Oostendorp, B.M. Zonderhuis, J.B. Tuynman

**Affiliations:** Department of Surgery, Cancer Center Amsterdam, Amsterdam UMC, Location VUmc, De Boelelaan 1117, Postbus 7057, 1007 MB, Amsterdam, the Netherlands

**Keywords:** Infected necrotizing pancreatitis, Necrosectomy, SILS, Minimally invasive technique, Case report

## Abstract

**Introduction:**

Approximately 5% of patients with acute pancreatitis develop infected necrotizing pancreatitis, with reported mortality rates up to 32%. Surgical interventions are postponed as long as possible, but if surgical debridement is needed the optimal approach has not been found yet.

**Case presentation:**

A 47-year-old male was referred to our tertiary centre with infected necrotizing pancreatitis. Two months after initial presentation and repeated percutaneous drainage, surgical retroperitoneal debridement of the necrotic tissue was performed using a single incision laparoscopic surgery (SILS) port. Postoperatively, percutaneous drainage was performed two more times, but no additional surgical interventions were needed. The patient was discharged one month after the surgical procedure.

**Discussion and conclusion:**

This is the first report of a minimally invasive technique using a SILS port for debridement of necrotizing pancreatitis. The ability to create a stable pneumo-retroperitoneum leads to optimal visualisation, better haemostasis, more space to operate in, better instrument handling, and better tissue control.

## Highlights

• 5% of patients with acute pancreatitis develop infected necrotizing pancreatitis.
• The best approach for debridement of necrotizing pancreatitis is under debate.
• Necrosectomy using a SILS port seems a good minimally invasive technique.

## 1 Introduction

Necrotizing pancreatitis occurs in 15% of patients with acute pancreatitis. Approximately one third of these patients develop infected necrotizing pancreatitis [1]. Which is defined radiologically as air configurations found in peripancreatic fluid collections, clinically by signs of infection as fever and increased inflammatory parameters, or by a positive culture following fine needle aspiration (FNA) [2]. Mortality rate of infected necrotizing pancreatitis is up to 32% [3].

The evidence-based guideline for the management of acute pancreatitis, published in 2013 by the International Association of Pancreatology (IAP) and American Pancreatic Association (APA), recommends a step-up approach starting with supportive care [2,4]. This includes fluid resuscitation, nutrition, pain medication, and in case of (suspected) infection of necrotizing pancreatitis intravenous antibiotics [5]. If a patient does not respond to conservative treatment or in case of clinical deterioration, an invasive intervention is required [6]. Intervention of first choice is to percutaneously drain the abscess. Percutaneous catheter drainage may prevent surgical necrosectomy in 23–50% of the cases [4]. However, in the majority a debridement of the necrosis is necessary to remove the source of ongoing infection [2]. It has been shown that early intervention, either percutaneous or surgical, is associated with poor outcomes. Therefore, it should be postponed until at least four weeks after onset if possible, preferably until after the necrosis has become walled-off (GRADE 1C) [2].

The optimal approach for surgical debridement of necrotizing pancreatitis is still under debate. Kokosis et al. have provided an overview of the different approaches that already exist, such as laparotomy, laparoscopy, retroperitoneoscopic, endoscopic and step-up approaches [7]. Frequent complications of major surgery are new-onset multiple organ failure, systemic sepsis, fistula, perforation of a visceral organ, intra-abdominal bleeding, new-onset diabetes and death [8]. Current less invasive procedures are associated with reduced morbidity, but at the cost of less effective debridement.

In order to reduce morbidity and improve patient outcome following surgical debridement, new methods should be explored. The patient was treated in an academic setting and we describe a novel minimally invasive approach for retroperitoneal access by using a single incision laparoscopic surgery (SILS) port and continuous insufflation. This offered improved visualisation and working space during the procedure compared to current minimally invasive approaches.

This case report has been reported in line with the improved SCARE checklist. The SCARE Guidelines were published in 2016 to provide a structure for reporting surgical case reports. In 2018, a modified and improved SCARE checklist was presented, after a Delphi consensus was completed [9].

## 2 Case presentation

A 47-year-old Caucasian male was referred to this hospital with an infected necrotizing biliary pancreatitis. On initial presentation at the emergency department of the referring hospital, a CT scan of the abdomen showed a pancreatitis with distension of the bile ducts. A concrement in the distal common bile duct was successfully removed through endoscopic retrograde cholangiopancreatography (ERCP), followed by a papillotomy. After ten days the patient was discharged.

**Fig. 1:**
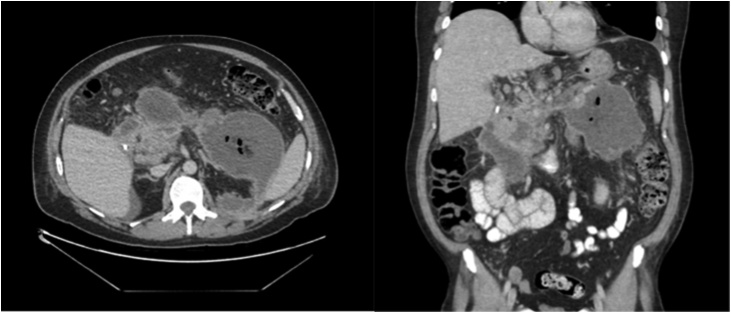
CT scan of the abdomen before drainage. This scan shows a pancreatitis and necrosis with air configurations in peripancreatic fluid collections.

Six days later, the patient was re-hospitalised due to progressive pancreatitis and necrosis on imaging with air configurations in peripancreatic fluid collections (*see* Fig. 1) suggesting infected peripancreatic collections [2,10]. Intravenous antibiotic therapy (meropenem) was started, which led to clinical improvement and decreasing CRP levels.

**Fig. 2:**
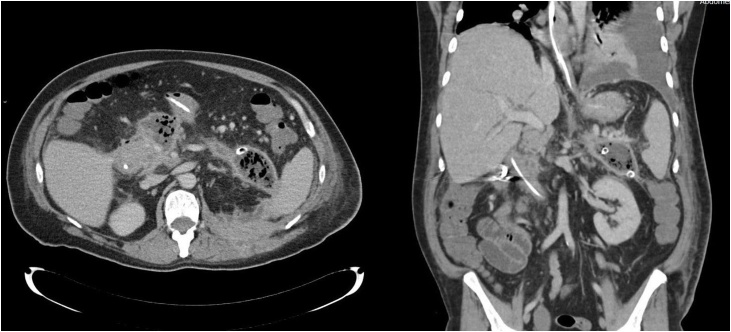
CT scan of the abdomen after drainage. This scan shows still the presence of a pancreatitis and ongoing necrotic tissue.

Conservative treatment was continued until 40 days after initial presentation of biliary pancreatitis. Due to minimal clinical improvement, peripancreatic fluid collections were drained by the radiologist with two percutaneous catheters (*see* Fig. 2). These were flushed with saline solution three times a day. Another 30 days later, 10 weeks after initial admission, a necrosectomy was deemed necessary due to lack of clinical progress.

## 3 Surgical approach

**Fig. 3:**
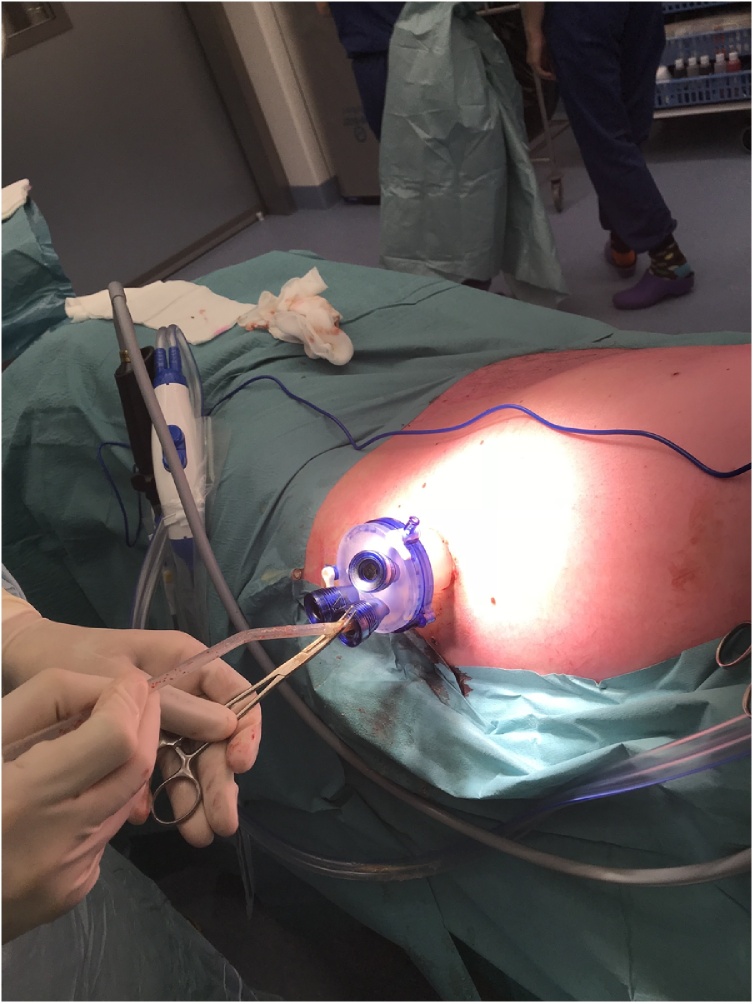
Application of the SILS port in the left lateral side of the patient.

**Fig. 4:**
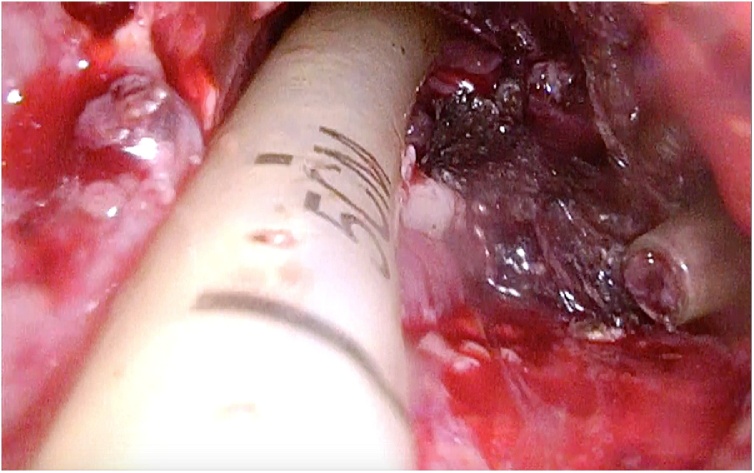
Drain-guided access to the necrotic tissue.

**Fig. 5:**
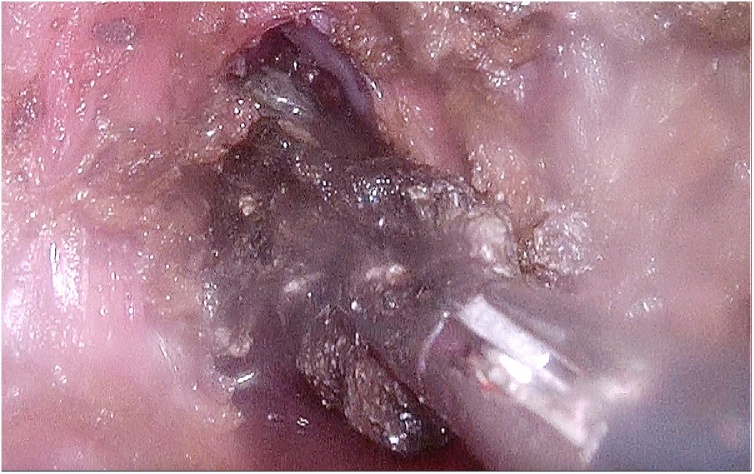
Minimally invasive debridement of necrosis.

The patient was positioned in supine position, tilted to the right lateral side, under general anaesthesia. The skin and subcutis were incised for three centimetres circumferencing one of the situated percutaneous drains. Once the trajectory was incised with conventional diathermy, we applied a SILS port (Gelpoint path Applied Medical) with Airseal® (Conmed) insufflator (*see* Fig. 3). By following the tract of the drain, the cavity containing the infected necrosis was reached. In the small cavity good vision and a stable retroperitoneal working space was enabled both by the 15−18 mmHg continuous insufflation and the 3D laparoscope with 30 degree angle (Olympus). All visual necrosis was debrided with use of laparoscopic instruments (see Fig. 4, Fig. 5 and the video (in Supplementary material)). After haemostasis, a new drain was positioned into the cavity and sutured to the skin.

Postoperatively the drain was flushed twice a day with 50cc sterile saline. Immediate after the operation, the patient did not develop any severe adverse events. Preoperatively, the level of CRP was 107 and the level of leukocytes was 16.8 and two days after the operation the level of CRP was 62 and the level of leukocytes was 15.3. Postoperative course required two additional percutaneous drainage procedures. Thereafter, the patient improved and was discharged one month after the surgical debridement.

## 4 Discussion

Currently, the preferred surgical approach is video-assisted retroperitoneal debridement (VARD), which we modified by making use of a SILS port to improve access to the retroperitoneal space. This system allowed us to create a stable pneumoretroperitoneum (15 mmHg), through which we accomplished better visualisation, more space, and better haemostasis during the procedure compared to our experience with a VARD approach. We assume that better visualisation and continuous pressure allow more optimal and complete debridement of the necrosis, and provide safety avoiding bleeding complications. VARD is often difficult and less precise due to limited exposure and suboptimal instrument handling, which is a substantial risk at bleeding and incomplete debridement.

Another advantage of this technique is the minimisation of the required incision length, which is smaller than required in VARD. An incision of approximately 3 cm is sufficient to insert the SILS port and continue the procedure laparoscopically. The single port provides a stable platform with better instrument handling, leading to better tissue control and the opportunity to ligate blood vessels. It also allows the surgeon to work with two instruments, instead of one instrument and a camera in VARD. All of the above advantages result in easier handling of the tissues with less manipulation of its surroundings. This might result in less postoperative pain, reduced superficial surgical site infections, and better wound healing. Hence, we impose this is a less minimal invasive technique than the widely performed VARD procedure.

A potential obstacle to implement this technique might be availability of the equipment, which might not be ready accessible in all hospitals. An investment is required in disposable SILS-ports and Airseal insufflation systems. However, once obtained, they could be used for a variety of minimally invasive procedures such as transanal minimally invasive surgery (TAMIS), including local excision, total mesorectal excision (TME) or other procedures [11]. Furthermore, the SILS technique is technically challenging and needs practice to accustom to the parallel instrument handling. Given the fact that surgical intervention is not routinely required in patients with acute pancreatitis, surgeons might need to gain experience with SILS in other procedures such as mentioned above. Nevertheless, for VARD and other approaches experience has to be gained in sequential interventions as well. By using SILS for different procedures, the learning curve of this technique might be shorter or similar.

## 5 Conclusion

This new surgical approach using a SILS port for retroperitoneal debridement provides a more effective and safe debridement of the necrosis due to potential benefits in terms of visualisation and operating space. We consider it as a more minimally invasive treatment compared to VARD for infected necrotizing pancreatitis.

## Declaration of Competing Interest

Dr J.B. Tuynman, N. den Dekker, A.A.J. Grüter, S.E. van Oostendorp and B.M. Zonderhuis have no conflict of interest.

## Sources of funding

This research did not receive any specific grant from funding agencies in the public, commercial, or not-for-profit sectors.

## Ethical approval

Waived by ethical committee of our institute.

## Consent

Patient signed informed consent for the use of clinical data, images of CT scan and video of surgical procedure.

## Author contribution

Nienke den Dekker and Alexander Grüter wrote the paper. Stefan van Oostendorp and Jurriaan Tuynman gave their feedback on the paper. Jurriaan Tuynman and Barbara Zonderhuis performed the surgical procedure.

## Registration of research studies

Not applicable, design is a case report.

## Guarantor

Jurriaan Tuynman (email address: j.tuynman@amsterdamumc.nl).

## Provenance and peer review

Not commissioned, externally peer-reviewed.

## Appendix A Supplementary data

The following video is supplementary data to this article:
